# Abrupt elevation of tumor marker levels in a huge splenic epidermoid cyst, a case report

**DOI:** 10.3389/fonc.2024.1415225

**Published:** 2024-06-25

**Authors:** Hao Cai, Zhenyu Hei, Guanghua Liu, Feng Zhao, Chunfeng Wang, Wenbin Guan, Gang Ren, Qing Zhou, Yi Dong, Ying Wang, Wei Gong, Litian Chen

**Affiliations:** ^1^ Department of General Surgery, Xinhua Hospital, Affiliated to Shanghai Jiao Tong University School of Medicine, Shanghai, China; ^2^ Shanghai Key Laboratory of Biliary Tract Disease Research, Shanghai, China; ^3^ Department of Transplantation, Xinhua Hospital, Affiliated to Shanghai Jiao Tong University School of Medicine, Shanghai, China; ^4^ Department of Interventional and Vascular Surgery, Xinhua Hospital, Affiliated to Shanghai Jiao Tong University School of Medicine, Shanghai, China; ^5^ Qianqiao Community Health Service Center, Shanghai, China; ^6^ Department of Pathology, Xinhua Hospital, Affiliated to Shanghai Jiao Tong University School of Medicine, Shanghai, China; ^7^ Department of Radiology, Xinhua Hospital, Affiliated to Shanghai Jiao Tong University School of Medicine, Shanghai, China; ^8^ Department of Oncology, Xinhua Hospital, Affiliated to Shanghai Jiao Tong University School of Medicine, Shanghai, China; ^9^ Department of Ultrasound, Xinhua Hospital, Affiliated to Shanghai Jiao Tong University School of Medicine, Shanghai, China

**Keywords:** splenic epidermoid cyst, tumor markers, CA19-9, CEA, CA125, laparoscopic splenectomy

## Abstract

Epidermoid cyst of the spleen is a rare disease, and relatively few cases were reported by literatures. Most published case reports provided inadequate information on the impact of splenic epidermoid cyst on tumor markers. A 32-year-old woman with a giant splenic epidermoid cyst was reported, for whom the serum concentration of a collection of tumor markers (CA19–9, CEA, CA125, CA242, and CA50) increased abruptly accompanied by left upper abdominal pain for 5 days. After comprehensive preoperative examination and multidisciplinary team discussion, we ruled out any concurrent malignancy and a laparoscopic total splenectomy was performed, during which the splenic cyst spontaneously ruptured unexpectedly. After surgery, the elevated serum tumor marker levels decreased sharply until reaching normal range 3 months later. Learning from the case, we conclude that interval monitoring of serum tumor markers is of critical value for patients with splenic epidermoid cyst. Abrupt elevation of tumor marker levels and abdominal pain may serve as signs of cyst rupture, which is strongly indicative of surgical intervention as soon as possible. Total removal of the splenic cyst is strongly suggested considering the recurrence and malignant potential of the splenic epidermoid cyst.

## Introduction

Cystic lesions in the spleen could be of various histological origins and could be divided into true cyst and pseudocyst, according to the presence of epithelial lining. Most splenic cystic lesions are asymptomatic and were occasionally found during routine health management. As a kind of true cyst, epidermoid cyst of the spleen is a rare disease and relatively few cases were reported by literatures. In addition, most of these reported cases provided inadequate information on the impact of splenic cyst on tumor markers (1).

This case report highlights the biological behavior of a splenic epidermoid cyst and details the dynamic changes of tumor markers before and after surgical intervention.

## Case description

A 32-year-old woman was admitted on 22/11/2023 for “4-year history of asymptomatic splenic cyst and 5-day duration of left upper abdominal pain”. During the 4-year follow-up period, the size of splenic cyst remained stable (approximately 10 cm × 8 cm), and serum tumor markers including carbohydrate antigen (CA)19–9, carcinoembryonic antigen (CEA), CA125, CA242, and CA50 were within the normal range until 5 days ago. No other symptoms were presented, and physical examination showed mild left upper quadrant abdominal tenderness.

**Figure 1:**
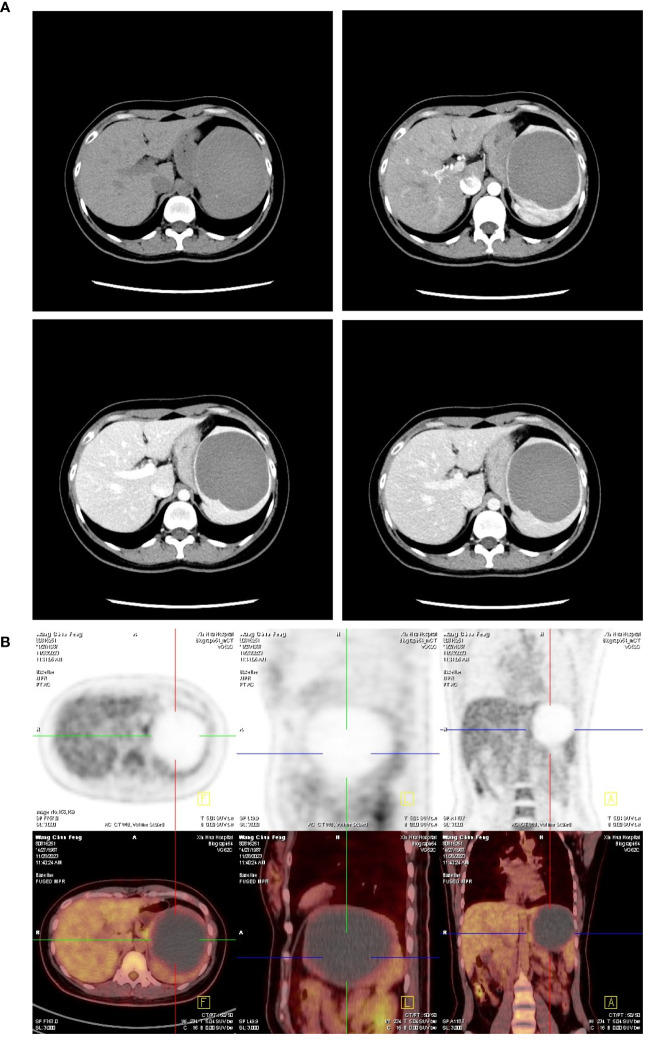
Abdominal imaging of the splenic cyst before surgery. **(A)** Representative four-phase image (non-contrast, arterial, portal, and liver phase) of the splenic cyst on contrast-enhanced CT scan showed no nodule or disproportionate thickening on the cystic wall except for tiny calcification was observed. There was also no contrast enhancement for the whole lesion constantly. **(B)** PET/CT scan showed no 18F-FDG uptake by the splenic lesion.

During the hospital stay, a routine blood test was performed. A collection of serum tumor marker levels were shown to be significantly elevated (CA19–9 7,109 U/ml, CEA 7.85 ng/ml, CA125 246 U/ml, CA242 >200 U/ml, CA50 >500 U/ml). To rule out concurrent tumor diseases, contrast-enhanced computed tomography (CECT) of the abdomen, positron-emission tomography and computed tomography (PET/CT) scan, and endoscopy examination were also performed. CECT and PET/CT scan showed that the CT value was 47 Hounsfield units within the cyst and tiny calcification was observed on the cystic wall without 18F-flurodeoxyglucose (18F-FDG) uptake for the whole lesion, which indicated that the splenic lesion might be a dermoid cyst or epidermoid cyst (**Figure 1**). Gastroscopy and colonoscopy showed superficial erosive gastritis and reflux esophagitis, ruling out possibly concurrent gastroduodenal and colorectal malignancies. After all the above examinations have been done, a multidisciplinary team (MDT) discussion session was held, and a consensus was reached that the elevation of these serum tumor marker levels was more likely to be a consequence of the splenic cyst itself. Total removal of the splenic cystic lesion was strongly suggested.

**Figure 2:**
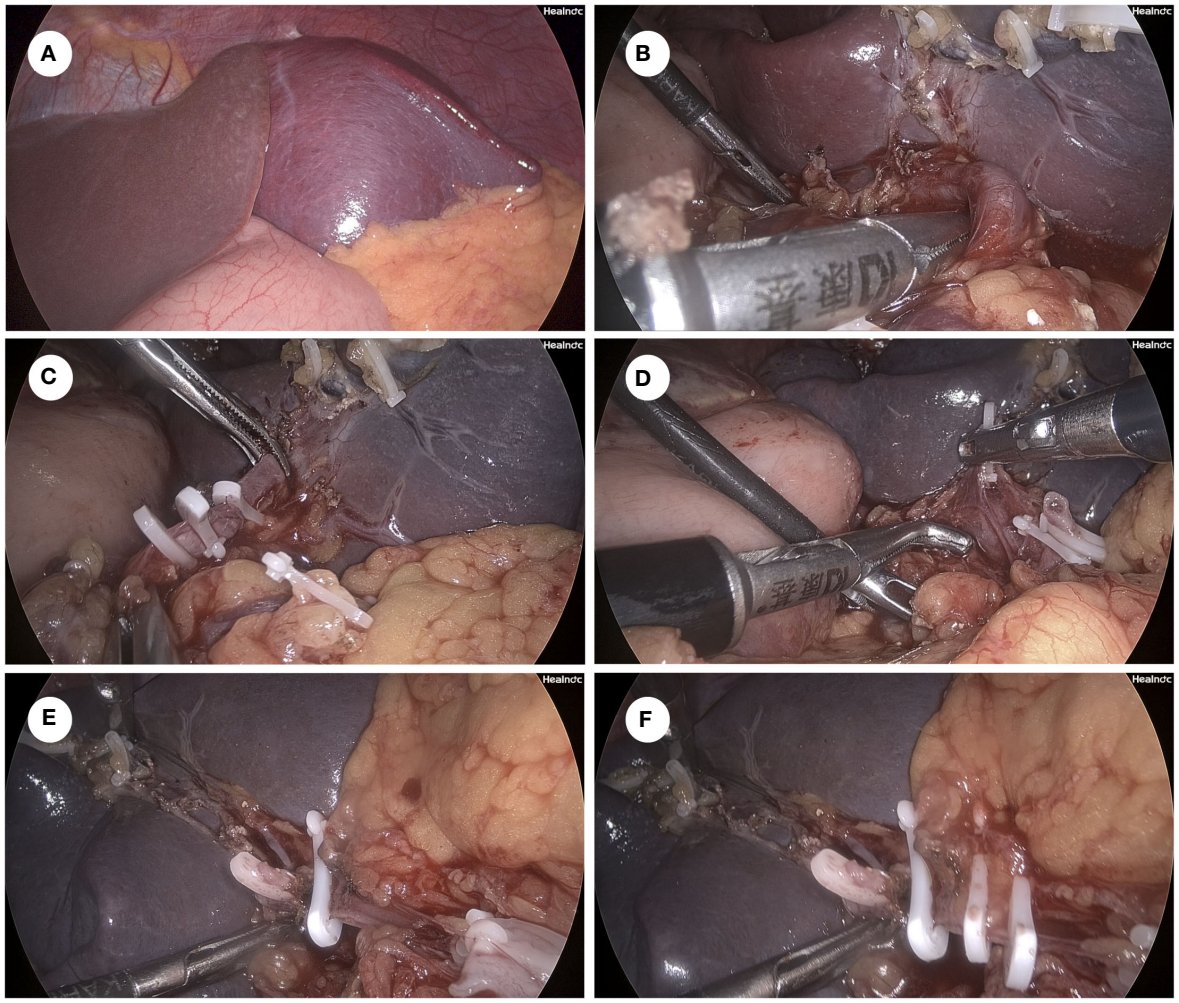
Key surgical procedure of laparoscopic total splenectomy. **(A)** Overview of the spleen and the splenic cyst on laparoscopy. **(B)** Dissociation of the splenic artery using the right-angle clamp. **(C)** Ligation of the splenic artery by using the Hem-O-Lock clips. **(D)** Dissociation of the splenic vein using the right-angle clamp. **(E)** Ligation of the to-be-dissected distal part of the splenic vein by using one Hem-O-Lock clip. **(F)** Ligation of the to-be-preserved proximal part of the splenic vein by using 2 Hem-O-Lock clips.

After informed consent was obtained from the patient, we performed a laparoscopic total splenectomy to ensure the complete removal of the splenic cyst on 04/12/2023. During the operation, the splenic cyst spontaneously ruptured as we tried to lift the left lateral liver lobe to better expose the splenic lesion. Dark-brown and turbid fluid spilled out of the splenic cyst, which was sampled for further examination followed by thorough suction. Perisplenic ligaments were carefully dissected, followed by the ligation and transection of the splenic artery and vein separately. After that, the total spleen was dissociated and placed into a specimen bag, where the spleen was cut into pieces and taken out of the abdominal cavity through a dilated trocar port incision (**Figure 2**). The surgical duration was 3 h, and intraoperative blood loss was 100 ml.

**Figure 3:**
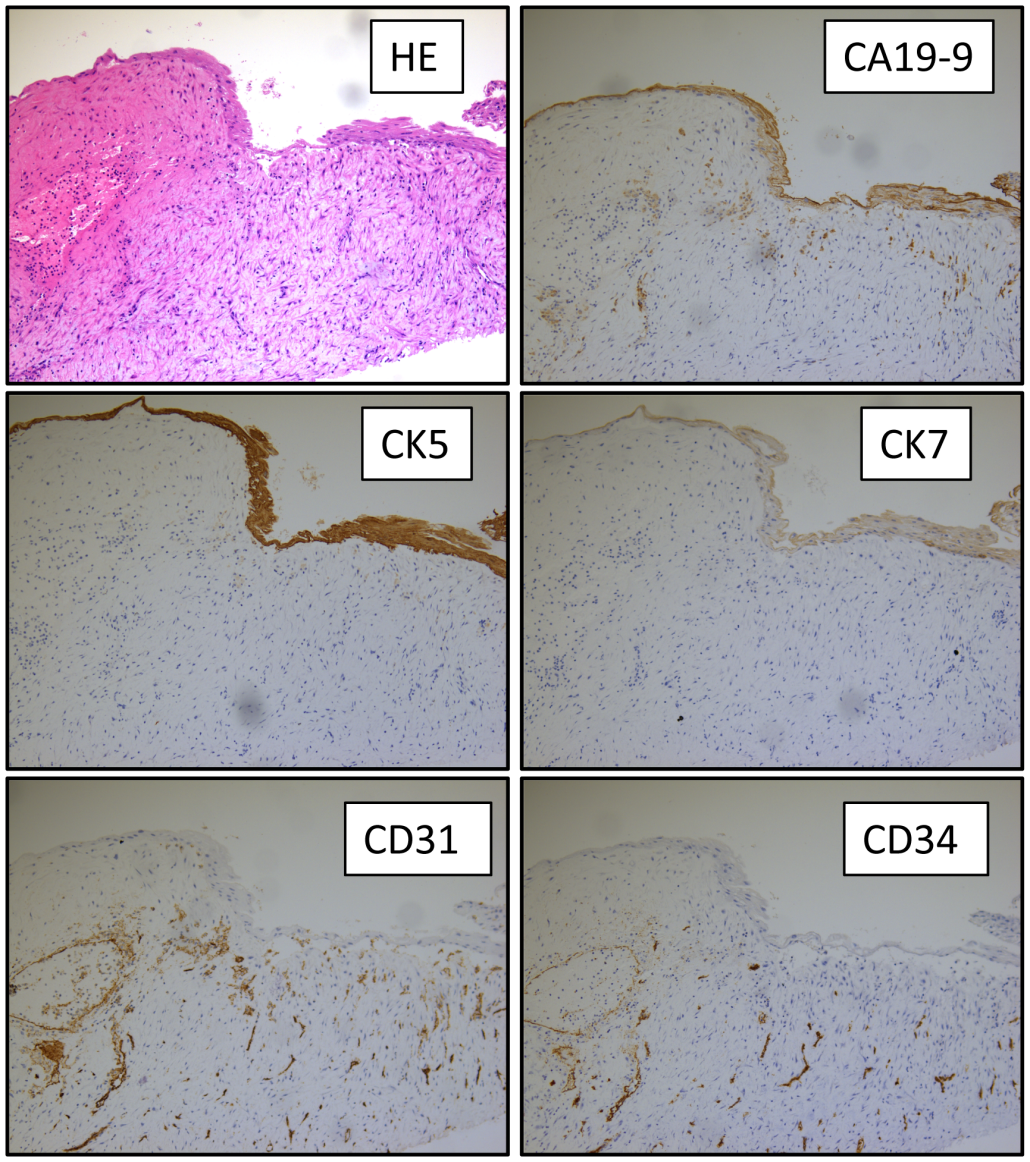
Pathological examination of the splenic cyst. HE and IHC (CA19–9, CK5, CK7, CD31, and CD34) staining of the splenic cyst capsule at 400× magnification.

Biochemical test of the cystic fluid showed that CA19–9 was >100,000 U/ml, CEA was 3,880 mg/ml, CA125 was >165,950 U/ml, CA242 was >200U/ml, and CA50 was >500 U/ml. The bacterial culture of the cystic fluid was negative, which ruled out infection within the splenic cyst. Pathological examination showed that the inner wall of the cystic capsule was lineated with simple cuboidal epithelium and squamous epithelium. Fibrosis and acute/chronic inflammatory cell infiltration was observed within the cystic capsule. Immunohistochemical (IHC) staining showed a positive expression for CK5, CK7 (partially), CD34, CD31, CA19–9, and Ki67 (5% on epithelium). The expressions of CEA, estrogen receptor (ER), and progesterone receptor (PR) were all negative (**Figure 3**). Therefore, a splenic epidermoid cyst was diagnosed histologically.

Prophylactic cephalosporin administration was prescribed to prevent infection after splenectomy. For this patient, daily injection of 4,100 iu low-molecule heparin was prescribed on the first day after surgery to prevent the formation of thrombosis within the portal system. Unfortunately, postoperative ultrasound scan showed mild thrombosis within the left and right branch of the portal vein. Therefore, we increased the dose of low-molecule heparin to 4,100 iu twice a day for 21 days, followed by the maintenance with rivaroxaban (15 mg p.o. qd). No other postoperative complication was presented.

**Figure 4:**
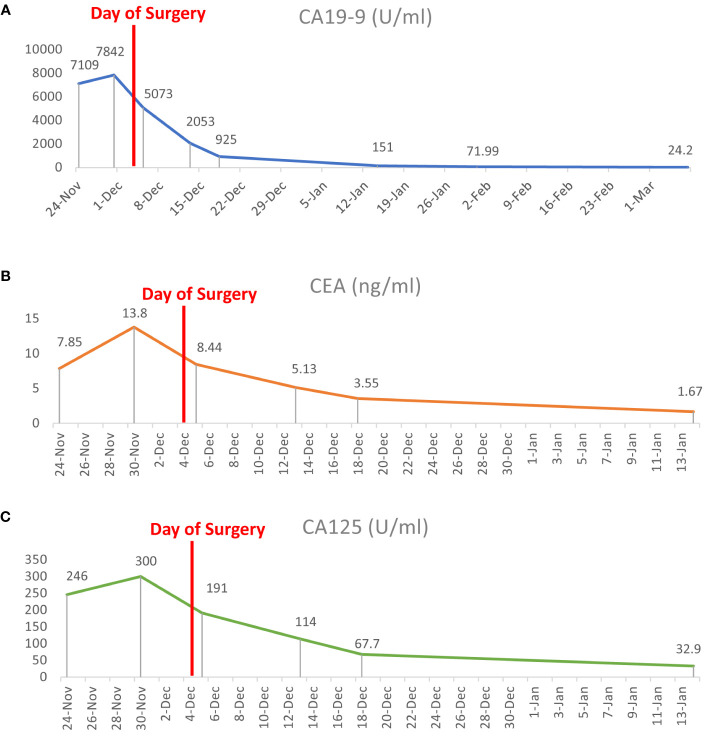
Dynamic changes of serum tumor marker levels before and after surgery. **(A–C)** The serum concentrations of CA19–9, CEA, and CA125 dropped quickly after laparoscopic total splenectomy until reaching the normal range 3 months later.

This patient was discharged 2 weeks after surgery and was routinely followed up for serum tumor markers and ultrasonography monthly, which showed that the abovementioned serum tumor marker levels declined steadily. The serum CEA level reached the normal range 1 month after surgery, followed by CA125, CA242, and CA50 levels after 1 more month, whereas the CA19–9 level reached the normal range after 3 months postoperatively (**Figure 4**).

This case report was approved by the ethics committee of Xinhua Hospital (approval number: XHEC-D-2024–044).

## Discussion

Splenic epidermoid cyst, dermoid cyst, and hydatid cyst are common types of true splenic cyst. It is difficult to tell them apart preoperatively, due to non-specific clinical manifestation and radiological features (2–4) (**Supplementary Table 1**). In this case, there was no personal history of parasite-endemic region settlement. Except for tiny calcification observed on the cystic wall, no cartilage, bone, snail, or hair tissue was found within the splenic cyst; Also, no daughter cyst was seen. Therefore, an epidermoid cyst in the spleen instead of a dermoid cyst or hydatid cyst was diagnosed. It has been reported that both epidermoid cyst and dermoid cyst could be accompanied by elevated CA19–9, CEA, or CA125 levels (5–10). The squamous epithelium cell component may be the source of secreted CA19–9 or other tumor antigens, as shown by IHC staining (11). However, not all cases reported the dynamic changes of tumor markers. Takagi et al. reported a case of epidermoid cyst within an intrapancreatic accessory spleen, which showed a sharp increase of CA19–9 before surgery and a normal serum CA19–9 level 4 weeks after surgical resection (12). Okuno et al. reported an elevation of serum CEA levels in a patient with ruptured splenic epidermoid cyst, which was normalized 3 months after decapsulation (8).

Usually, the significant abnormality of serum tumor marker levels is highly indicative of malignancies, which needs further examination to confirm diagnosis. Elevated CA19–9 is suspicious of malignancies arising from the biliary tract or pancreas; elevated CEA is a sign of possible alimentary tract cancer; and elevated CA125 is less specific and may be indicative of tumors involving multiple tissues including the female reproductive system. For this patient, however, preoperative examinations ruled out any concurrent malignant tumor diseases. Interval surveillance of tumor markers had been normal for the past 4 years. A high concentration of tumor markers in this cystic fluid and a sharp decline of serum tumor marker levels after surgery further confirmed that these tumor markers were sourced from the splenic cyst. Malignant transformation has been reported in an epidermoid cyst in intrapancreatic accessory spleen, for whom preoperative PET/CT scan also showed increased 18-FDG intake on the cystic wall with a slight increase of serum CA19–9 and normal CEA levels (13). Similarly, there might also be a malignant potential for patients with splenic epidermoid cyst.

It has been reported that serum tumor marker levels are positively correlated with the size or the symptom of splenic cyst (14, 15). In this patient, the size of the splenic cyst was consistent before and after serum tumor marker levels started to increase. No fever was presented, and blood tests for inflammation were normal before surgery. The serum concentration of tumor markers increased quickly after the patient was presented with left upper abdominal pain, which may indicate a sign of rupture of the splenic cyst (16). Before surgery, we rechecked the tumor markers to rule out any possible error in the lab test, which showed that these tumor marker levels were even higher than those on admission. Subsequently, the splenic cyst spontaneously ruptured very easily during the operation. Microorganism test of the cystic fluid ruled out concurrent bacterial infection within the lesion. On the basis of the above evidence, we speculated that the tension within the splenic cyst started to increase 5 days before admission, approaching the critical point of rupture for unknown reason, causing left upper abdominal pain and squeezing the intra-cyst tumor antigens to enter the blood stream. Therefore, early surgical intervention is necessary to prevent the spontaneous rupture and possible intra-abdominal bleeding outside the hospital, which may be a life-threatening emergency for the patient.

Decapsulation is not suggested since recurrence has been reported after decapsulation of the splenic epidermoid cyst (17). Also, since there might be a malignant potential for epidermoid cyst (13), complete removal of the splenic lesion is strongly recommended. Partial splenectomy for splenic dermoid cyst has been reported (18), which may have the advantage of preserved splenic function; however, it also increases the risk of postoperative intra-abdominal hemorrhage and ischemia of remnant spleen. Pitiakoudis et al. reported a case of splenic cyst undergoing extended laparoscopic partial decapsulation. However, total splenectomy was performed 5 days later due to possible splenic ischemia (19). For the patient of our case report, as the splenic cyst took most part of the total spleen, future remnant splenic volume after partial splenectomy was so small that the blood supply could not be assured. Therefore, total splenectomy is relatively safer than partial splenectomy, although it increases the risk of postoperative portal thrombosis. Portal vein thrombosis is a major modality after total splenectomy due to high platelet count and decreased blood flow from the splenic vein. After considering the pros and cons of partial splenectomy, this patient chose to remove the total spleen for fearing the relatively higher risk of postoperative hemorrhage or ischemia and unplanned secondary surgery. For this case, postoperative portal thrombosis was identified, even after the administration of low-molecule heparin. Ultrasound scan showed mild thrombosis within the left and right branches of the portal vein; strikingly, no thrombus was detected within the splenic vein or superior mesenchymal vein. After strengthening anticoagulant therapy, the portal thrombus was almost totally resolved and smooth portal blood flow was restored 1 month after discharge, indicating that the risk of portal thrombosis after total splenectomy is controllable and partially reversable.

## Conclusion

For patients suspicious of splenic epidermoid cyst, interval monitoring of serum tumor markers is of critical value. In case of abdominal pain accompanied by abruptly elevated tumor marker levels, rupture of the splenic cyst could happen in near future and surgical intervention should be considered as soon as possible. Total removal of the splenic cyst is strongly suggested considering the recurrence potential of the splenic epidermoid cyst.

## Data availability statement

The original contributions presented in the study are included in the article/**Supplementary Material**. Further inquiries can be directed to the corresponding authors.

## Ethics statement

The studies involving humans were approved by Ethics Committee of Xinhua Hospital Affiliated to Shanghai Jiao Tong University School of Medicine. The studies were conducted in accordance with the local legislation and institutional requirements. Written informed consent for participation was not required from the participants or the participants’ legal guardians/next of kin in accordance with the national legislation and institutional requirements. Written informed consent was obtained from the individual(s) for the publication of any potentially identifiable images or data included in this article.

## Author contributions

HC: Writing – original draft. ZH: Writing – original draft. GL: Writing – review & editing. FZ: Writing – review & editing. CW: Writing – review & editing. WBG: Writing – review & editing. GR: Writing – review & editing. QZ: Writing – review & editing. YD: Writing – review & editing. YW: Writing – review & editing. WG: Writing – review & editing. LC: Writing – review & editing.
