# Supplementary material for: Abrupt elevation of tumor marker levels in a huge splenic epidermoid cyst, a case report
Source: Front Oncol. 2024 Jun 25;14:1415225. doi: 10.3389/fonc.2024.1415225 (PMC11231066; doi:10.3389/fonc.2024.1415225)
Supplement: Supplementary file 1 [file Table_1.docx]

**Supplementary Table 1 Summary of the clinical manifestation and radiological features of common splenic cysts**

|  | Clinical manifestation | Radiological features |
| --- | --- | --- |
| Splenic hydatid | Non-specific (asymptomatic, abdominal mass, abdominal pain, or symptoms caused by compression of adjacent organ) | Simple cyst with thin wall; Detached germinative membrane; Daughter cysts; Focal Calcification |
| Splenic epidermoid cyst |  | Simple cyst with thin wall; Focal Calcification |
| Splenic dermoid cyst |  | Multiple tissue component; Heterogeneous density; Focal Calcification |
